# Integrating Lean Six Sigma into Microbiology Laboratories: Insights from a Literature Review

**DOI:** 10.3390/healthcare13080917

**Published:** 2025-04-16

**Authors:** David Sancho, Antonio Rezusta, Raquel Acero

**Affiliations:** 1 Instituto de Investigación en Ingeniería de Aragón (I3A), 50018 Zaragoza, Spain; racero@unizar.es; 2 Design and Manufacturing Engineering Department, University of Zaragoza, 50018 Zaragoza, Spain; 3 Microbiology Unit, Miguel Servet University Hospital, 50009 Zaragoza, Spain; arezusta@salud.aragon.es; 4 Instituto de Investigación Sanitaria de Aragón (IISA), 50009 Zaragoza, Spain

**Keywords:** healthcare, microbiology laboratory, clinical laboratory, lean six sigma, efficiency, quality

## Abstract

Background/Objectives: Clinical laboratories are fundamental to healthcare systems, contributing to over 70% of clinical decisions while accounting for only 2–3% of hospital budgets. Among them, microbiology laboratories provide critical information that directly influences patient outcomes and satisfaction. This study presents a structured review of the current state of Lean Six Sigma (LSS) implementation in microbiology and comparable laboratory environments. The objective is to identify relevant contributions within the state of the art to highlight potential benefits applicable to microbiology laboratories and to detect persistent gaps and unresolved needs. Methods: A systematic literature review was performed across six databases (Web of Science, ScienceDirect, Scopus, ProQuest, PubMed, and Google Scholar) to identify studies published between 2012 and September 2024. After screening, 33 studies were selected for full-text analysis. Results: The selected literature was analyzed to assess the extent to which LSS methodologies have been applied in microbiology laboratories. Particular attention was given to the definition and use of key performance indicators (KPIs). While industry-adapted metrics such as cost reduction and turnaround time are commonly employed, clinical indicators, such as patient impact, satisfaction, and diagnostic accuracy, are underutilized. Additionally, the analysis revealed a frequent omission of the control phase in LSS projects, limiting long-term process monitoring. The review also identifies the most suitable LSS tools and evaluates how laboratories manage interruptions in routine workflows. Conclusions: Future research should prioritize the integration of clinical KPIs into LSS frameworks, establish robust control phases for sustained monitoring, and systematically address the impact of process interruptions on optimization efforts.

## 1. Introduction

Lean Six Sigma (LSS) has demonstrated high usability in optimizing processes in the industry, for example, by reducing wastes, such as unnecessary transportation, excess inventory, unnecessary human motion, increased waiting time, overproduction, and overprocessing, and by reducing defects, meaning that the result of the process does not meet the necessary requirements and needs to be corrected or repeated. The method continues to be improved and has grown enormously in many sectors [1].

Originally, LSS was the combination of two methodologies: Lean and Six Sigma, both designed for the manufacturing industry. Toyota developed the Lean methodology in the 1940s under the original name Toyota Production System (TPS). The goal of this method was the optimization of production systems by reducing seven types of wastes [2]. Later, Motorola developed Six Sigma, in the 1980s, with a focus on reducing variation using a system with a four-stage problem-solving process: Measure, Analyze, Improve, and Control–MAIC. Afterward, a step called Define was added, creating the current DMAIC method. LSS is a methodology widely used and continuously improved, with growth sustained [3] due to the successful results in process optimization across industries like manufacturing [4], food [5], aerospace [6], automotives [7], and others. Since its beginning, with the expansion of Lean techniques, pioneers in these techniques have quickly realized the potential benefits for healthcare [8].

Regarding application areas in healthcare, numerous examples demonstrate the widespread application of the Lean and LSS methodologies to optimize various health sectors [9,10,11,12,13,14]. For example, LSS is used in multiple healthcare areas, such as patient management in hospitals [15] to reduce costs, for patient waiting times [16,17], and for length of stay [18] Also, it has been deployed in surgical processes [19] or enhance efficiency in some specialties like ophthalmology [20]. It also reduces patient waiting times in trauma orthopedics [21] and improves efficiency in urology [22] and oncology [23].

Despite the increasing interest in LSS techniques within healthcare and their potential benefits in optimizing clinical laboratory processes—particularly in microbiology—there is currently no comprehensive review that examines the state of the art, recent advancements, and critical factors for implementing LSS in microbiology laboratories. Key aspects such as relevant KPIs and the most suitable tools for deployment remain unexplored. Consequently, many claims are based on practices from other laboratory types, whose applicability and effectiveness in microbiology require further analysis.

LSS Healthcare adapted the tools and key performance indicators (KPIs) from the industry to healthcare. Typical KPIs used include cost reduction, customer satisfaction, financial savings, and increased production capacity [24], but in this case, the focus is on the patient. Therefore, new KPIs must be defined, such as patient waiting time and satisfaction, hospital length of stay, process turnaround time, and patient safety, among others. The application of LSS has proven to have a significant impact on the healthcare sector by reducing costs and improving the quality of patient care, including reducing preventable deaths [25].

Therefore, it is extremely important to define specific KPIs that will focus not only on process optimization, but also on patient impact, directly linking to clinical decisions and relevant clinical parameters. Questions related to the outcomes of final treatment, mortality, or quality of life improvement need to be addressed in these types of projects. On these grounds, how the patient’s health outcomes are linked to common KPIs measured in LSS projects gains relevance, and we pose the following research question:

RQ-1.—How are KPIs determined, and what data are utilized to select the KPIs and tools for LSS healthcare projects?

Regarding LSS tools, it is quite common to use only one or two isolated LSS tools to optimize a process without implementing a full LSS project following the DMAIC structure [26,27,28] These projects are focused on increasing the performance of a process [29] or creating an optimized layout during a change such as automation implementation, measuring the performance of changes [30] Additionally, optimizations are sometimes evaluated with a pilot project [31,32]. However, in some cases, the complete DMAIC process is not completed, and the control phase is not documented or performed, although some studies highlight the lack of monitoring and control as one of the most common causes of LSS project failure [33]. In healthcare, the project’s analysis and control in the mid-long term is crucial due to the impact on the patient, with the control phase being very important in ensuring the real impact of the optimization. Therefore, the second research question we would like to raise is the following:

RQ-2.—Do the LSS healthcare projects described in the literature use the control phase as part of a DMAIC process? How important is this stage in healthcare?

One of the most interesting areas in which to apply LSS in healthcare is hospital clinical laboratories [34]. In terms of importance, clinical laboratories provide information for more than 70% of clinical decisions [35]. However, despite this importance, some articles state that the budget of the laboratory accounts for only 2–3% of total healthcare expenses in most hospitals. The impact of clinical or microbiology laboratories on the patient has been widely demonstrated [36,37]. For example, laboratory response time significantly affects the morbidity and mortality of infections caused by multidrug-resistant bacteria [38]. The microbiology laboratory, due to its characteristics, has significant potential for process optimization, as it involves many manual procedures. Laboratory process automation is relatively recent, not widely implemented, and not used in all types of cultures. This results in a complex and highly variable workflow that depends on the type of sample and the suspected pathogen. Additionally, there are long waiting times due to the need for sample incubation, making it essential to maximize efficiency so that waiting periods can be utilized for other tasks, or be planned strategically to minimize major disruptions to relevant processes. Additionally, the improvement of laboratory workflows is especially relevant due to the use of critical samples such as blood cultures. Optimizing sample prioritization by selecting patients who would benefit from rapid pathogen detection [39] improves survival rates in severe sepsis, as it enables earlier administration of the appropriate antibiotic treatment [40]. Both the laboratory results’ delivery time and their accuracy play a relevant role in the process and have a high clinical impact, as was stated in [41] for microbiology laboratories.

Considering patient impact in the healthcare sector, improving only the process is not always sufficient; sometimes, the LSS project reveals that the process is not meeting the best clinical requirements. In the case of critical microbiology cultures such as blood cultures, measuring solely production KPIs transposed to healthcare is not enough to analyze the suitability of the process. Other clinical factors must be considered, such as delivery time from extraction to incubation, incubation time, blood volume, etc. [42]. Optimization of the entire project must consider both KPI optimization (result delivery time, patient waiting time, etc.) and clinical requirements to maximize result reliability. Therefore, RQ3 assists in gaining an overview of this important requirement.

RQ-3.—Are the clinical parameters a part of the LSS optimization process?

Finally, it is important to note that healthcare processes are normally affected by a high number of interruptions and exceptions. This can be observed in microbiology laboratories, where process interruptions occur daily. For example, a sample may need to be prioritized due to a request from another section due to the patient’s severity or due to unexpected results found in complementary medical tests. This generates uncertainty in the process, making interruptions and exceptions in healthcare processes not as predictable as in a manufacturing environment. Methods of managing these unexpected process variations efficiently in LSS projects so as not to negatively impact relevant process KPIs need to be considered, leading to the final research question, RQ4, analyzed in this work.

RQ-4.—Is there any standardized system implemented for managing interruption and priorities inside clinical laboratory processes?

This work aims to leverage these questions and other supplementary information to identify the most pertinent contributions within the current state of the art, ultimately extracting insights with direct applicability to microbiology laboratories. By analyzing results obtained not only in microbiology but also in other comparable laboratory settings, it becomes possible to highlight immediate, transferable benefits that could improve the laboratory’s efficiency, accuracy, and overall performance. Additionally, this approach allows for the identification of persistent challenges and unresolved needs that have not yet been sufficiently explored, thereby informing future lines of research and practical implementation strategies.

The information detailed in the objective is presented and methodically organized in the following manner. Section 2 provides the methodological foundation of this review and explains the selection of keywords used, filters, and exclusion criteria to obtain the selection of articles to be analyzed in the next steps. Section 3 presents the literature review findings, classified by different standardized criteria. Section 4 presents a discussion analysis of the different review findings that can provide the information requested in the research questions, and what this work brings new to existing literature. Finally, Section 5 concludes this paper with contributions, implications, and a future research agenda.

## 2. Methodology

The literature review was conducted following the authors’ recommendations in [43] and PRISMA guidelines [44,45,46] to ensure the analysis of the maximum number of articles using an appropriate classification methodology for optimal information extraction. The keywords were selected to ensure maximum coverage of the related topics. In this step, language and publication date filters were applied. Next, subject or research area filters were used to ensure that the results obtained were linked to the theme in scope (microbiology laboratory or related laboratories such as clinical or pathology among others). Finally, a manual review of the abstract and article content is necessary to determine whether the article contributes relevant information to the research. In addition to the methodology applied following the previous recommendations, a quality appraisal was conducted using the CASP checklist to evaluate aspects such as study validity, methodology, and other key factors. The checklist is available in the Supplementary Materials.

The search was finished in September 2024; therefore, papers published after this date have not been included in this review. The query was launched in six of the most used search engines. Five of them, Web of Science, Science Direct, Scopus, ProQuest, and PubMed, have been selected due to the size of the database and the filters available that allow us to fine-tune the results and optimize the number of analyzed articles in the manual filter stage. The sixth search engine is Google Scholar; in this case, the filters are not as advanced as the other search engines, and manual filtering is needed. However, Google Scholar could offer different results compared with others due to the indexing method used in the database, so it has been used to ensure all articles available on the topic have been analyzed.

The keywords selected were divided into three groups (Table 1), moving from less to more specific. This methodology has been used to avoid limitations in the results of the search due to the keywords.

Group 1: Core Search Keywords: Lean laboratory and DMAIC laboratory are the keywords used, giving a high number of results. Filtering by intensive use of the area or subject is needed to obtain an affordable number of results.

Group 2: Generic Related Topics: This is more specific than Group 1, where the keywords cover any type of laboratory. In Group 2, the focus is on the Lean and DMAIC methodologies related to healthcare and clinical laboratories, establishing a health-related context for the search.

Group 3: Specific Topic: the keywords are specific, focusing on microbiology laboratories, so the filters need not be as intensive, and resulting in very accurate outcomes based primarily and only on the keywords.

The initial search yielded 4693 articles. Of them, we considered only articles published after 2012 and written in English or Spanish, reducing the total to 3223 articles. Next, we reduced the results further using the area or subject filters of the search engine (for example, excluding topics like oil, material manufacturing, chemical industry, etc.), which totaled 2056 articles to be filtered by screening their titles, reducing the entries to 227. These entries are not unique references, so the duplicates were removed to obtain 103 articles that would be analyzed manually by reading the abstract and content. These filtering procedures were divided into stages with different criteria (explained in Table 2), adding transparency to the selection of relevant studies.

**Figure 1:**
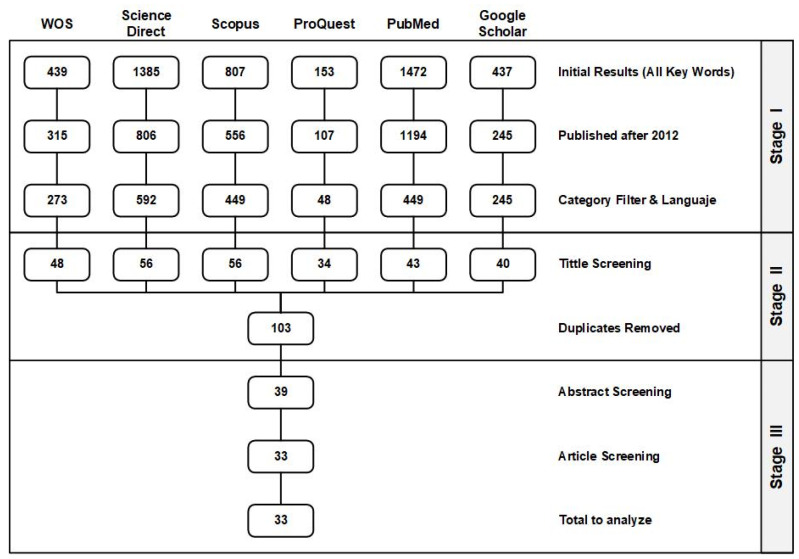
Review process—article filtering stages.

The filtering process described in Table 2 resulted in the articles represented in Figure 1 for each search engine. As explained above, in Stage I, only the final articles were considered, suppressing all preprints or editorials, reducing the number of articles from 4693 to 2056. After that, the methodology explained was conducted to reduce the number of articles to 39 articles after Stage II. In the selected literature, in Stage III, the abstract and the article content were revised to obtain the final 33 articles to be analyzed in the review and to extract the information needed to analyze the current state of the art and for the analysis of the research questions.

## 3. Findings and Results

The results have been analyzed in stages, increasing the level of detail. From an initial number of 4693 papers, the screening process resulted in 2056 papers for Stage I, 103 papers for Stage II, and finally 33 papers for Stage III.

### 3.1. Stage I. Initial Results, Publication Year, Language, and Subject Filters

**Figure 2:**
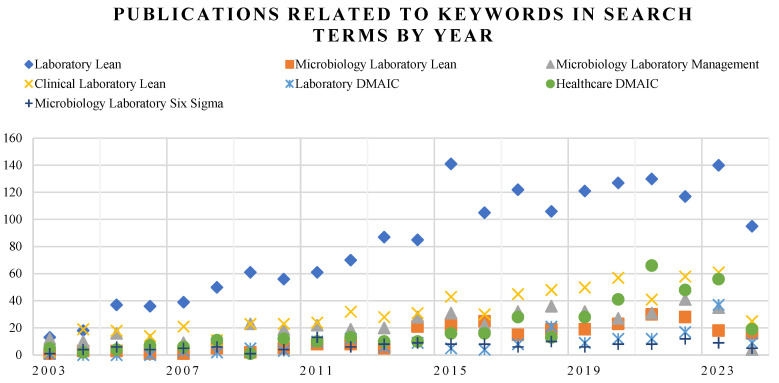
Publications related to keywords in search terms by year.

In the first search stage, we analyzed the articles containing the search terms in Table 1, and as expected, depending on the keyword used, the most generic terms showed more results than the more specific ones. Figure 2 represents the results of articles published from 2003 to October 2024, with all keywords adding only one filter to remove preprints.

The core search term Laboratory Lean generated the biggest number of search results, 2277, with increasing figures of publications since 2003, but included lean practices applied to any type of laboratory, and would require a posterior area filtering to obtain a quantity of articles affordable to analyze manually by reading the abstract and the text. The second core search term Laboratory DMAIC offered a lower number of search results throughout the period (126).

Generic terms search like Healthcare DMAIC have a high number of results (371). Although more focused on the health environment, these terms need to be filtered by subject. The second generic search term “Clinical laboratory lean” shows an increasing number of related publications (809).

The specific topic keywords are Microbiology Laboratory Lean, with 347 articles, Microbiology Laboratory Management with 675, and 88 articles on Microbiology Laboratory Six Sigma. The specificity of the keywords implies a reduction in the overall results; however, the final number of articles to analyze manually was closer to the initial number because the results were more precise, and the filters did not reduce the final number of articles. In any case, the trend over the years is increasing, clearly visible with the core term laboratory Lean.

**Figure 3:**
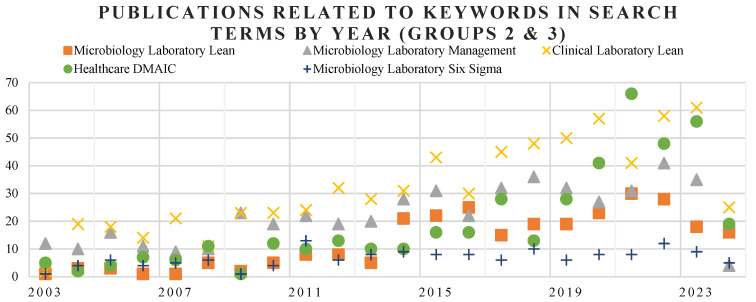
Publications related to keywords of Group 2 and 3 in search terms by year.

**Figure 4:**
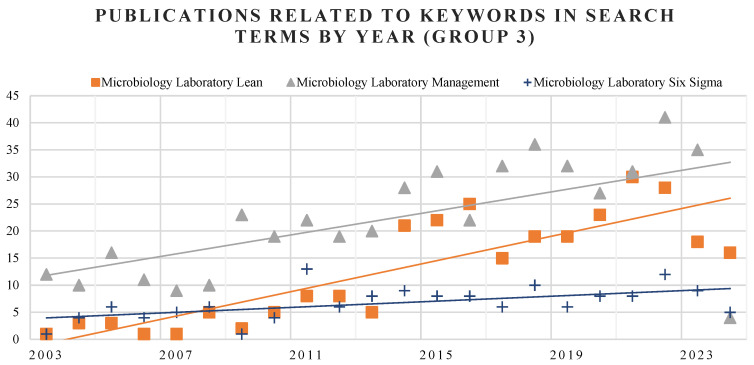
Publications related to keywords of Group 3 with trendline added.

To enhance data presentation and interpretation, individual graphs with a reduced scale have been generated, as shown in Figure 3. This graph focuses on specific aspects of the dataset to provide clearer insights and highlight key patterns. Figure 3 presents results based solely on the keywords from Groups 2 and 3. An increasing number of publications related to the application of Lean in clinical laboratories is observed from 2012 onward, whereas papers concerning Healthcare DMAIC began to rise approximately five years later. Focusing on the context of microbiology laboratories, as depicted in Figure 4, the highest number of publications is related to general laboratory management. Lean practices applied specifically to microbiology labs rank second, followed by the deployment of DMAIC methodology, which ranks third.

**Figure 5:**
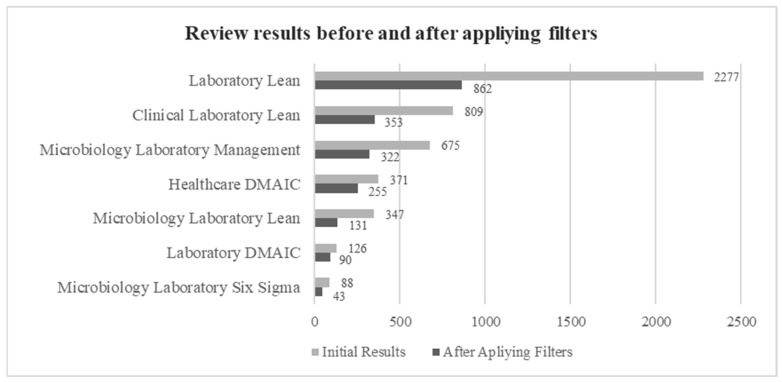
Total results by keywords before and after applying filters (publication date >2012 and subject/area).

After implementing the language filter and publication date after 2012, the number of papers was reduced from 4693 to 3223, cutting the results by approximately 30%. The last filter in Stage I is the subject or area filter of the search engine, which further reduces the number of articles to 2056. In Figure 5, the initial results are compared with those obtained after applying filters. The reduction in results for generic terms like “Laboratory Lean” is very high compared with the most specific, like “Microbiology Laboratory Sigma”, due to the specificity of the terms. In this case, the filtering has been performed manually by reading the title and abstract. In all cases, the keywords are chosen to ensure that the search engines retrieve all articles related to the application of LSS in the microbiology laboratory.

### 3.2. Stage II: Title Screening

The objective of this review is to analyze the extent of LSS methodologies and tools applied to microbiology laboratories. In this stage, the titles of the articles were read manually to reduce the results from 2.056 to 257 articles. The keywords used in title screening have been chosen to obtain a list of articles that could contain information about the LSS tools and KPIs used, among others. Duplicates were removed before further analysis, resulting in 103 articles whose abstracts and contents were analyzed in detail to make the final classification and extract the necessary information.

### 3.3. Stage III: Abstract and Article Analysis

In the last filtering stage, the content of the articles is deeply analyzed. In most cases, the abstract provides details about the methodology and KPIs used, allowing for initial filtering that reduces the results from 103 to 39 articles. Then, a detailed content analysis was conducted, leading to a final selection of 33 articles.

**Figure 6:**
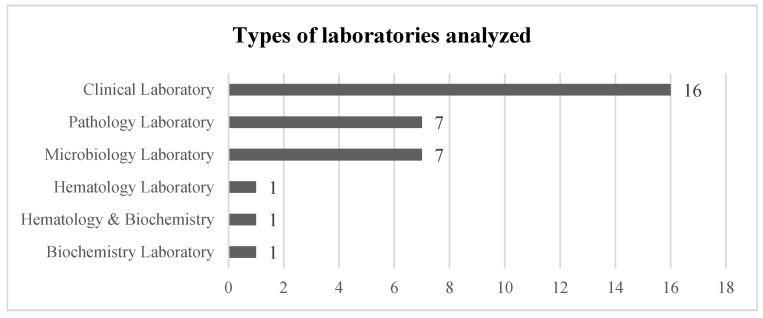
Types of laboratories analyzed in selected articles from the review.

The review process focuses on LSS techniques applied to microbiology laboratories. According to the inclusion criteria, several factors have been analyzed, such as the type of laboratories where LSS projects are deployed in the healthcare sector, the main LSS tools used in these projects, and KPIs selected for progress monitoring. The type of laboratories analyzed in the papers was the first inclusion criterion considered. The scope of this review is the microbiology laboratory, although the analysis carried out in this type of lab is not always performed in a specific laboratory; some hospitals, mainly small ones, perform all work in clinical laboratories. In addition to clinical and microbiology laboratories, Figure 6 shows publications referring to LSS projects applied to other related laboratories in healthcare facilities.

**Figure 7:**
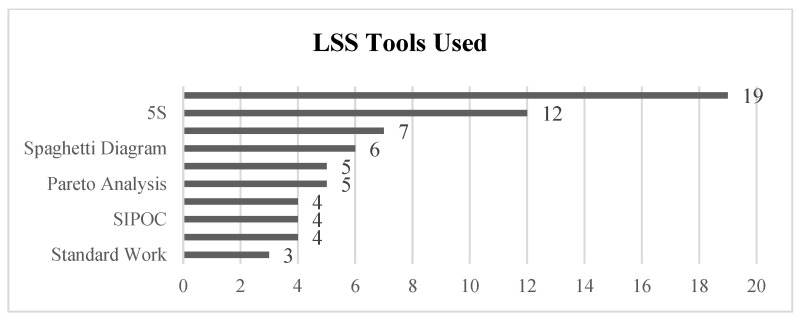
Top 10 tools used in selected articles from the review.

Secondly, we centered our research on the LSS tools cited in the papers selected in Stage III. Analyzing whether the tools commonly used in the manufacturing sector are also applied to LSS projects in healthcare, and specifically in laboratories, offers valuable insights. In Figure 7, the number of articles including the application of the top 10 LSS tools are listed. The most common tool identified in the research is Value Stream Mapping (VSM), used in 19 articles, followed by 5S in 12 publications. The third position is held by the DMAIC methodology, used in 7 articles, compared to the second tool, used in 12. Other LSS tools that are used less frequently include Spaghetti Diagrams, waste reduction analysis, Pareto analysis, CTQs, SIPOC, fishbone diagrams, and standard work.

Another important aspect to consider is whether the LSS projects identified in the literature are complete DMAIC projects, or if some of their phases are omitted. The research showed that most of the articles do not cover a complete LSS project. In most cases, a partial LSS tool application is carried out to optimize a process by removing waste, and reducing the takt-time or cycle time using VSM [27,47,48,49,50,51,52]. 5S is the second most used tool, and its application is sometimes derived from a previously performed process analysis [26,28,53,54,55]. Waste analysis [56] or spaghetti diagrams [30] are easy-to-use tools that produce very good results in the short term. To analyze how many articles optimize a laboratory through a comprehensive LSS project, employing multiple tools to achieve a comprehensive optimization, rather than focusing on isolated aspects of the process, the DMAIC methodology is commonly used. In this context, DMAIC ranks third in importance in Figure 7 among the tools identified.

**Figure 8:**
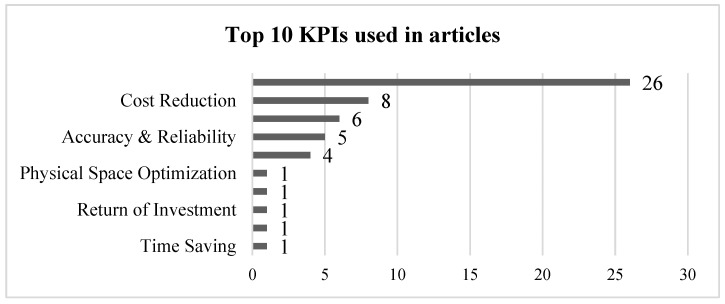
Top 10 KPIs measured in selected articles from the review.

In Stage III of the review, the next concept examined was the definition and application of *KPIs* in LSS projects within clinical and microbiological laboratories. To analyze the results of an LSS project, the objectives must be first defined by measurable KPIs that will be followed before and after the LSS project to analyze the real process improvement. In Figure 8, the top 10 KPIs identified in the final screened articles are shown. The most used KPI is turnaround time, mentioned in 26 of the 33 final articles selected [26,30,57]. The second most frequent KPI is cost reduction, cited in 8 articles [47,52,54]

Linking this result to the usage of the VSM tool in the first position, we can expect that the most used KPI would be turnaround time, an indicator normally used to measure the process lead-time in VSM applications. This KPI is optimizable with other very commonly used tools like Spaghetti Chart (by reducing travel time) or 5S, among others. Optimizing a process and reducing the turnaround time allows for improvements to additional indicators, included in the publications represented in Figure 8, by increasing the laboratory testing capacity [52], optimizing resources such as physical space optimization [58] and enabling cost reduction [53,59]. This KPI also has clinical impact because of the reduction in the workflow lead-time. For example, in sepsis, reducing the time needed to adjust the treatment based on blood culture analysis results has a significant effect on mortality rates [60]. Reducing turnaround time has also other positive effects in other relevant KPIs, such as the patient length of stay in the hospital and the derived costs [61,62].

Although the most common indicator across all studies is turnaround time (tied with patient waiting time in the case of pathology laboratories), the second most important indicator varies depending on the type of laboratory analyzed, as shown in Table 3. In clinical and microbiology laboratories, the second most analyzed KPI is cost reduction. However, in microbiology laboratories, patient waiting time is tied in first place, whereas in clinical laboratories, it ranks fourth, behind the analysis accuracy. This could indicate that, depending on the type of laboratory, certain process optimization tools may be more effective in improving one KPI over another. Additionally, due to their specific characteristics, some laboratories may prioritize certain KPIs over others.

Another key finding from the review regarding KPIs is that most indicators do not focus on their impact on the patient. Although patients may be affected indirectly, only a few articles have analyzed the direct impact of the LSS implementation on the patient, focusing solely on production-related indicators like turnaround time or test capacity (production capacity), among others. The KPIs identified in the review process that could directly influence the patient are waiting time [29,48,49,50,63,64] and length of stay in the hospital [65]. However, in these cases, the real impact in the mid- or long-term, measured through variables such as mortality and medical consequences, is not analyzed.

**Figure 9:**
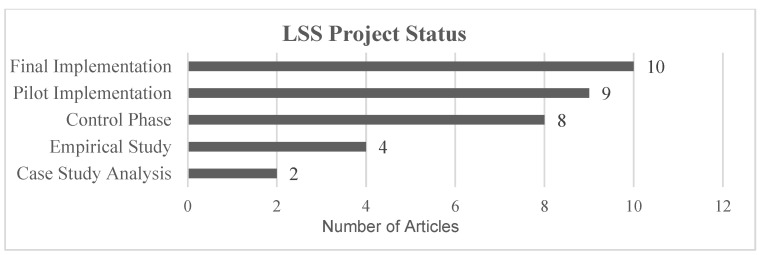
Degree of implementation of LSS projects in selected articles from the review.

Finally, the articles are classified by the degree of implementation of the LSS project in the real environment. The implementation level provides valuable information about the data obtained in the real world with unforeseen events that occur during the pilot test or time after the final implementation. Figure 9 shows five different study classifications depending on the degree of implementation of the LSS project.

- Empirical Study: the theoretical study of the advantages of LSS, or methodology implementation for a specific field.
- Case Study: an optimization problem is identified in a real environment, presenting the article as a theoretical analysis of LSS tools application to improve the process.
- Pilot Implementation: the process analyzed in the article has been optimized in a real environment and KPIs were measured, but no plan has been established for implementation as a permanent workflow.
- Final Implementation: a permanent optimization workflow was established after process analysis.
- Control Phase: the project was implemented in a real environment, with processes in place to detect deviations in KPIs. In some cases, the author outlined techniques to correct these deviations.

Most articles described LSS projects applied in a real laboratory environment totaling 27, while others were theoretical or without implementation. Projects that led to permanent changes to workflow are classified in the articles as being in the final implementation and control phase, with 18 out of the 33 articles in this category. In contrast, 9 of the 33 articles that focus on pilot projects provide no information on final implementation or whether the project led to permanent process changes. This classification is important for identifying unforeseen events during or after the optimization process and the mid/long-term effects in a real environment after project implementation.

Articles with research content not applied in a real environment are classified as empirical studies [54,66,67,68] and case study analysis [51,69] which represent 18% of the total. Therefore, most of the articles filtered are LSS projects applied in a real environment.

All the references reviewed and analyzed in Section 3 of this review are listed in Appendix A. This appendix includes relevant information for our research, such as the year of publication, LSS tools used, KPIs, laboratory type, and project status achieved in the study.

## 4. Discussion

After filtering and classifying the articles, they were analyzed again to address the research questions. These questions were selected after reviewing articles about LSS applied to healthcare and insights gained from the experience of the authors in optimization projects carried out in a microbiology laboratory where certain needs were detected together with clinical stakeholders. This section outlines the contribution of our study, organized as answers to the four sets of questions posed in the introduction.

RQ-1.—How are KPIs determined, and what data are utilized to select the KPIs and tools for healthcare LSS projects?

Most LSS projects in healthcare are designed using the same methodology as manufacturing optimization projects, without considering specific requests in the healthcare context. KPIs commonly used in manufacturing are cost reduction, testing, or production capacity, normally related to productivity increase [70]. The first seeks cost decrease and the second links directly with turnaround time because, in most cases, resources are fixed, so if the turnaround time decreases, capacity could increase.

It has been identified in the literature review that usually the main goal of the LSS project is to reduce the process turnaround time, but only a few articles link TAT optimization with the real impact on the patient. This impact could be measured with indicators such as patient satisfaction or patient waiting time, as noted in some studies [55]. Reviews analyzing the impact of this feature due to his importance [71]. In other articles, the benefits of reducing patient waiting time have been assessed by measuring the derived increase in bed availability and enhancing patient safety [64]. Other examples proved waiting time reduction to be a factor contributing to increased patient satisfaction [49].

Focusing on clinical and microbiology laboratories, it is generally assumed that the importance of microbiology laboratory results increase speed and reliability in decision-making across most departments, especially in emergencies [72] or in intensive care units (ICUs) with patients with severe pathologies such as sepsis [73]. Some authors suggest that the turnaround time is a reliable indicator of laboratory effectiveness for all types of samples: routine and emergency [74]. Some reviews revealed that patient impact could also be indirectly influenced by optimizing KPIs like error reduction, or the above-mentioned turnaround time. The patient impact is also measured using the waiting time as a KPI analyzed from various perspectives, including the urgency of the sample and its optimization in clinical laboratories [48] or reducing biochemistry laboratory congestion [29].

The rejection rate due to contamination is an important KPI in microbiology laboratories [75,76]. Contamination of various samples, such as blood culture [77,78] and urine cultures [79] is a common quality issue that must be monitored. However, the review did not identify LSS projects deployed in microbiology laboratories measuring the sample contamination rate as a KPI. The sample rejection rates measured are those related to lack of documentation, ineligible samples, and other factors [28].

Therefore, it is crucial to establish well-defined KPIs that not only emphasize process optimization, but also assess patient outcomes by directly linking to clinical decision-making and relevant clinical parameters. This consideration is particularly significant in microbiology laboratories, which are the focus of our study. In LSS projects applied to clinical laboratories, it is essential to address questions related to final treatment outcomes, mortality rates, and improvements in patients’ quality of life. This may require large-scale data analyses from hospital and laboratory databases, enabling retrospective assessments of the implementation of optimization measures derived from LSS projects in the laboratory and their impact on key indicators such as mortality rates, length of hospital stay, and treatment effectiveness. Additionally, they could be compared with the limited literature available on patient impact in other disciplines like surgery [80].

RQ-2.—Do the projects described in the literature use the control phase as part of a DMAIC process? How important is this stage?

In general, the articles analyzed focus on optimizing specific indicators using individual LSS tools, such as Value Stream Mapping (VSM) (see Figure 7), more than using a global project approach. These tools are typically applied in isolation rather than being integrated into a comprehensive LSS optimization project following a DMAIC cycle. Typically, the DMAIC structure is used to analyze a complete service or section to look for process optimization or quality improvement, for example, through the processes of a pathology laboratory [63], complete laboratory analysis optimization and error reduction [59], and the simplification of laboratory processes by focusing on reducing turnaround time by eliminating non-value-adding stems [81] In other cases, a KPI requires optimization without a specific process or quality factor identified, so the DMAIC methodology helps to pinpoint strengths and weaknesses to improve a KPI, like patient waiting time, in a pathology laboratory [64] and to determine the best methodology for process quality enhancement.

A low percentage of articles (24%) use the control phase, and in many cases, this phase is not integrated into a DMAIC structure; see Figure 9. Among the articles that include a control phase, more than 57% are in the final or pilot implementation stage but do not use a control phase to systematically review the results.

Focusing on the application of the DMAIC process, sometimes the process is not completed, with the control phase either undocumented or omitted. Based on our review, only 7 of 33 articles use the complete DMAIC methodology, as is stated in Figure 7. In these cases, the projects were implemented in a real environment, and specific techniques are in place to detect deviations in KPIs and correct deviations in case they may be needed. Some articles use the control phase of the DMAIC to ensure that the process is correctly implemented and stabilized after the test or pilot period. This helps to keep KPIs optimized for the long term, but it requires documenting and standardizing procedures to detect deviations and ensure process stability [82]. Another similar practice is implemented in [59] for a clinical laboratory by comparing target KPIs before and after process optimization, and by monitoring the stability of the process over time, comparing customer requirements with the actual results. Other examples that implemented a control system to assure the stability of the process over time are [27,30]. Here, the authors implemented continuous KPI monitoring to ensure that urine, vaginal, and stool culture process indicators were within the established limits [30], or compared the KPI variation over time to ensure the impact of the project optimization in the patient was as expected [55].

The lack of indicator monitoring and control phase is one of the most common causes of LSS project failures. In healthcare, mid- to long-term project analysis and control are essential due to their impact on patient outcomes. The conducted review indicates that few LSS improvement projects applied to clinical laboratories include a full implementation of the DMAIC cycle with a control phase to assess the effectiveness of implemented measures. Instead, most projects remain limited to partial applications of the cycle or adopt approaches that are more theoretical. This makes the control phase particularly important for ensuring the sustained effectiveness of optimization efforts. Additionally, this is a crucial factor to consider in the design of LSS optimization projects applied to clinical laboratories, given the impact of diagnostic and sample result validation processes on the patient.

In healthcare process optimization, the control phase is crucial. However, it is even more important to use clinical KPIs to measure the impact on patients in the medium and long term. Ongoing monitoring over time in the control phase ensures the stability of the process, making the outcomes of different stages predictable. This allows for adjustments based on variations in clinical KPIs and helps to anticipate performance issues before they arise, thereby minimizing their impact on the patient. Therefore, a control phase needs to be implemented in all LSS projects with complete implementation after pilot test deployment. Relevant clinical KPIs need to be identified and monitored during this phase, defining a follow-up time long enough to ensure the stabilization of the new process and the tracking of the indicators.

RQ-3.—Are the clinical parameters a part of the LSS optimization?

In LSS projects applied to clinical laboratories, pre-analytical, analytical, and post-analytical processes are analyzed and optimized. In the pre-analytical phase, a relevant aspect to consider is the potential delay from sample extraction to processing, because it affects key clinical parameters. This is especially relevant to critical cultures requiring timely processing such as blood cultures [83], cerebrospinal fluid [84], urine [85], sputum [86] and [32]. In these cases, clinical parameters need to be considered carefully in the definition and monitoring of related LSS projects. For pre-analytical and analytical processes, it is essential to consider sample management and storage conditions from extraction to delivery. This is especially relevant for critical cultures in the microbiology laboratory to ensure the result’s reliability. Storage temperature affects fecal sample reliability [87], and sample volume impacts blood culture sensitivity [88,89] and other parameters.

Despite the emphasis placed by various medical case studies and research articles on the importance of clinical parameters in the processing of cultures and samples in microbiology laboratories, not all LSS studies identified in the review applied to clinical laboratories considered these parameters in the project definition. The lack of monitoring of key parameters—such as sample volume or the time elapsed from collection to processing—may prevent the detection of optimization opportunities. Consequently, this could reduce the accuracy of laboratory results and negatively influence patient outcomes. It would be advisable to consider clinical parameters in the definition of the LSS project.

RQ-4.—Is there any system for managing interruption and priorities inside the process?

In manufacturing processes, interruptions are typically caused by unexpected events such as equipment breakdowns, supply chain disruptions, and system failures. In the healthcare system, interruptions are frequent and often necessary for various reasons, including sudden changes in disease severity, the admission of a critically ill patient, and the discovery of an unexpected result in a sample.

Despite their importance, none of the reviewed studies have specifically addressed the management of interruptions in clinical laboratories. This is particularly relevant in microbiology laboratories, where diagnostic workflows are often disrupted by urgent cases, abnormal findings, or critical physician requests. The most common process analysis applied in laboratory workflow follows a First In, First Out (FIFO) approach [32], which does not account for real-time interruptions. As a result, when an urgent situation arises, there is no structured mechanism to temporarily halt the ongoing process, address the emergency, and then efficiently resume the previously interrupted workflow.

Moreover, interruptions frequently occur when an unexpected result is detected during sample analysis or when clinicians request expedited processing for a critical patient. These deviations from the standard FIFO workflow create inefficiencies, as they require manual intervention and disrupt routine operations. The absence of a systematic method for tracking and managing these interruptions leads to unoptimized process resumption, introducing variability and potential errors. Additionally, since these disruptions are rarely quantified, their impact on KPIs such as turnaround time, diagnostic accuracy, and resource utilization remains largely unknown.

The lack of structured interruption management is a common issue in the industry as well. Since the LSS methodology does not consider interruptions as part of the process, they are regarded as process instabilities that must be corrected.

In clinical laboratories, however, interruptions are an inherent part of the process, highlighting a critical gap in process optimization strategies. If interruptions can be identified and classified because they often appear in the standard process, process optimization can be applied by identifying the best phases in the standard workflow for managing these interruptions and designing standard procedures for interruption management and their integration in the standard workflow.

Future research should focus on developing interruption-resilient workflows, integrating real-time monitoring systems, and implementing adaptive scheduling techniques to ensure interruption management, maintaining laboratory efficiency, and without compromising urgent patient care. A possible research suggestion could be to adapt tools like Value Stream Mapping (VSM) to identify waiting times that are not eliminated through process optimization. These waiting times could then be strategically integrated into the workflow, allowing the process to be paused and interruptions to be introduced as a controlled sub-process. This approach would help minimize the negative impact of unexpected disruptions, making them a managed component of the overall system rather than an obstacle to efficiency.

This review has several limitations. Despite the use of multiple search databases and the broad time frame of the included articles, there may still be boundaries. Articles published after 2012 were selected to avoid conflicts between older techniques and the most recent advancements in the field. However, this could exclude some articles published before 2012 that include relevant information. In terms of language, only articles in English and Spanish were analyzed, and unfortunately, articles in other languages could not be included. The most significant limitation, however, is the lack of information identified in the research on the application of Lean Six Sigma methodologies in microbiology laboratories. To mitigate this, studies from similar clinical laboratories were included, where techniques could be extrapolated, but the volume of relevant articles remains limited due to the novelty of the topic.

## 5. Conclusions

LSS applied to the healthcare sector and microbiology laboratories is a compelling topic that has attracted research interest over the years. This methodology is widely used to optimize processes using different LSS tools or as part of a complete optimization project with a Six Sigma DMAIC structure.

The definition of the KPIs is a critical part of most LSS projects, not only to track the process improvement, but also to measure the impact on the patient. Usually adapted from manufacturing, most projects used indicators such as turnaround time, laboratory capacity or cost reduction, assuming that the patient impact is evaluated after optimizing the transposed manufacturing KPIs.

Therefore, other clinical valuable approaches and clinical parameters relevant to the pre-analytical, analytical, and post-analytical processes in the laboratory need to be considered in LSS projects, especially in the case of critical samples. The impact of LSS projects on the patient needs to be assessed after process optimization, including the clinical perspective. Useful clinical indicators such as length of stay, complications, 30-day mortality rate, and revisit rate could be considered and included in the analysis. The broad use of these and other similar KPIs integrated into control phases will allow us to compare the benefits of LSS process optimization in terms of patient impact. Big data techniques applied to Six Sigma project tracking and monitoring the impact on the patient could be valuable approaches to address.

Clinical factors are rarely considered in most of the articles analyzed despite their healthcare or clinical laboratory context. Therefore, in our opinion, future research should focus on analyzing the clinical implications at each step of LSS optimization process to ensure the reliability of the results and maintain consistent patient care.

The use of the DMAIC cycle and individual LSS tools is well documented in the literature, yielding highly positive results. However, in some cases, crucial phases—such as the control phase—are not implemented, making it impossible to track project indicators. The safety and the improvement of patient care need to be guaranteed and monitored. A valuable approach for LSS projects in clinical laboratories would be consistently including a control phase following the pilot implementation of improvement measures and carefully selecting the LSS tools that best align with the project’s context.

Finally, research on process optimization in clinical laboratories should explore the impact of interruption management on process optimization, focusing on two key areas: the adjustments required to address the interruption, and the steps necessary to effectively resume the main process.

Therefore, all these aspects require further research and the development of new tools or methodologies to fully realize the benefits that LSS methodology can bring to microbiology laboratory processes.

## Figures and Tables

**Table 1:** List of search terms for the systematic literature review.

**Core Search Terms (Group 1):**
Laboratory Lean Laboratory DMAIC
**Generic Related Topic (Group 2):**
Healthcare DMAIC Clinical Laboratory Lean
**Specific Topic (Group 3):**
Microbiology Laboratory Lean Microbiology Laboratory Management Microbiology Laboratory Six Sigma

**Table 2:** Article inclusion and exclusion criteria.

	Inclusion Criteria	Exclusion Criteria
Stage I	(+) Articles containing the search terms; (+) Articles in Spanish or English; (+) Relevant to healthcare: laboratory, healthcare, microbiology, pathology, etc.	(−) Preprint and editorials; (−) Published before 2012; (−) Not relevant to healthcare.
Stage II	(+) Article titles containing references to Lean, Six Sigma, or Lean Six Sigma about clinical laboratories.	(−) Article titles do not contain references to Lean, Six Sigma, or Lean Six Sigma applied to clinical laboratories; (−) Articles duplicated.
Stage III	(+) Articles abstract with scope on Lean, Six Sigma, or both in a clinical laboratory; (+) Articles including LSS tools or methodology.	(−) Articles without a clear LSS methodology, KPIs, or tools.

**Table 3:** Top 5 measured KPIs used by laboratory type (frequency of appearance in literature review).

	Turnaround Time	Cost Reduction	Patient Waiting	Accuracy and Reliability	Test Capacity
Biochemistry Laboratory	1		1		1
Clinical Laboratory	14	4	2	3	1
Hematology and Biochemistry	1				
Hematology Laboratory	1				
Microbiology Laboratory	6	3		1	1
Pathology Laboratory	3	1	3	1	1

## Supplementary Materials

The following supporting information can be downloaded at https://www.mdpi.com/article/10.3390/healthcare13080917/s1, Critical Appraisal Skills Programme (CASP) Checklist.

## Appendix A

**Table A1:** Papers selected with LSS tools, KPIs, type, and project status.

	Title	Year	LSS Tools Used	KPIs (Improved Value)	Laboratory Type	Project Status
1	“Utilization of 5S Visual Management: A Lean Six Sigma Application in Operation of Hospital Clinical Laboratory” [26]	2013	- 5S	- Turnaround time (improved >25%) - Cost reduction (revenue +30%)	Clinical Laboratory	Final Implementation
2	“Adoption of lean principles in a high-volume molecular diagnostic microbiology laboratory” [27]	2014	- Value Stream Map - Waste reduction	- Turnaround time (>85% results in <24 h) - Cost reduction (not specified)	Microbiology Laboratory	Control Phase
3	“Adoption of lean tools in medical laboratory industry: A case study of Namibia” [54]	2017	- Standardized work - PDCA - 5S - Value Stream Map	- Turnaround time (N/A—Empirical Study)	Clinical Laboratory	Empirical Study
4	“Application of lean management systems in pathology laboratory work process and laboratory environment” [28]	2019	- 5S - Value Stream Map	- Rejection sigma level of the sample (from 3.61 to 3.9) - Turnaround time (improved by 30%) - Process time (improved by 67%)	Pathology Laboratory	Pilot
5	“Applying lean flows in pathology laboratory remodeling” [47]	2014	- Spaghetti diagram - Value Stream Map	- Turnaround time (not specified) - Cost reduction (not specified) - Time saving (eliminated 16.25 min per day shift) - Test capacity (increased by 20%)	Pathology Laboratory	Final Implementation
6	“Applying Lean methodologies reduces ED laboratory turnaround times” [90]	2015	- Spaghetti diagram - Value Stream Map	- Turnaround time (reduced up to 12) - Cost reduction (reduced $62.400/year)	Clinical Laboratory	Final Implementation
7	“Conversion of a classical microbiology laboratory to a total automation laboratory enhanced by the application of lean principles” [30]	2024	- Spaghetti diagram - Process mapping - Time and Motion - A3 - 5S - Standardized work	- Turnaround time (reduced up to 89%) - Test capacity (increased by 124%)	Microbiology Laboratory	Control Phase
8	“Experience of lean six sigma quality approach to hospital laboratory services” [48]	2015	- Value Stream Map	- Patient waiting time - Check-in time - Number of queue abandonments - Number of front-office check-ins (not specified)	Clinical Laboratory	Pilot
9	“Externalities of lean implementation in medical laboratories. Process optimization vs. adaptation and flexibility for the future” [67]	2021		- Turnaround time - Accuracy and reliability of results (not specified)	Clinical Laboratory	Empirical Study
10	“Impact of lean interventions on time buffer reduction in a hospital setting” [55]	2017	- 5S - Pareto analysis - Variability reduction	- Turnaround time - Variability (not specified)	Clinical Laboratory	Control Phase
11	“Improvement of laboratory turnaround time using Lean methodology” [57]	2018	- 5S - Value Stream Map - Pareto analysis	- Turnaround time (reduced up to 10 min)	Hematology and Biochemistry	Final Implementation
12	“Improvement of urgent tests laboratory turnaround time through laboratory lean management” [56]	2020	- Waste analysis - Visual control - Standardized work - 5S - Kanban	- Turnaround time (reduced by 4 min)	Clinical Laboratory	Final Implementation
13	“Improving the efficiency of the center for medical biochemistry, clinical center ni[, by applying lean Six Sigma methodology” [29]	2014	- Waste analysis - 5S - Value Stream Map - Pareto analysis	- Turnaround time (reduction >45%) - Patient waiting time (reduction up to 75%) - Reducing congestion in the laboratory	Biochemistry Laboratory	Pilot
14	“Integrating Lean and Automation for Enhanced Serology Diagnosis Efficiency in Tertiary Healthcare Microbiology Laboratories” [52]	2024	- VSM	- Turnaround time (Reduction up to 87.3%) - Cost reduction (reduced >7.1%) - Test per day (66% more test per day)	Microbiology Laboratory	Final Implementation
15	“Lean healthcare as a tool for improvement: A case study in a clinical laboratory” [49]	2017	- Value Stream Map	- Patient waiting time (not specified)	Clinical Laboratory	Pilot
16	“Lean methodology for pathology laboratories: A case study from a public hospital” [63]	2019	- Value Stream Map - Fishbone diagram - Pareto analysis - 5S - Visual management - Standardized eork	- Patient waiting time (improvement of 4.6%) - Turnaround time (reduced by 67%)	Pathology Laboratory	Pilot
17	“Lean Six Sigma in Laboratory Process” [59]	2023	- DMAIC - SIPOC - CTQ - Pareto analysis - Fishbone diagram - Whys analysis - Value Stream Map	- Cost reduction (reduction of 40%) - Turnaround time (reduction of 40%)	Microbiology Laboratory	Control Phase
18	“Lean six sigma methodologies improve clinical laboratory efficiency and reduce turnaround times” [81]	2018	- DMAIC	- Turnaround time (Reduced by 9 min) - Reducing errors (Reduced from 30% to 3%)	Clinical Laboratory	Final Implementation
19	“Lean thinking in hospital: Case study at the pathology laboratory” [91]	2015	- Waste analysis - Spaghetti diagram - Value Stream Map	- Takt time - Error minimization (not Specified)	Pathology Laboratory	Pilot
20	“Lean-Agile adaptations in clinical laboratory accredited ISO 15189” [53]	2015	- 5S	- Cost Reduction (2 technicians fewer) - Turnaround time (no variation after cost reduction)	Clinical Laboratory	Control Phase
21	“Optimizing the Supply Chain of Intensive Care Unit Blood Culture Samples to Clinical Microbiology Laboratory Using Lean Six Sigma” [83]	2023	- Waste analysis - Value Stream Map - Process activity analysis	- Turnaround time (reduced >55%)	Microbiology Laboratory	Final Implementation
22	“Preliminary results of Lean method implementation in a pathology lab from Northeastern Brazil” [50]	2015	- Value Stream Map - Spaghetti diagram - A3	- Patient waiting time (reduced 55%) - % of correct and completed task. (improved 122.2%)	Pathology Laboratory	Pilot
23	“Providing critical laboratory results on time, every time to help reduce emergency department length of stay: How our laboratory achieved a six sigma level of performance” [65]	2013	- Automation with LSS philosophy	- Turnaround time - Patient length of stay - Return of investment (not Specified) - Productivity (improvement of 35%)	Clinical Laboratory	Final Implementation
24	“Reducing patient waiting time in a pathology department using the Six Sigma methodology” [64]	2013	- DMAIC - CTQ - SIPOC - C&E diagram - PCA - Gemba Walk	- Patient waiting time (reduced by 50%)	Pathology Laboratory	Control Phase
25	“Reducing the turnaround time of laboratory samples by using Lean Six Sigma methodology in a tertiary-care hospital in India” [69]	2020	- DMAIC - CTQ - SIPOC - Value Stream Map - I-MR - Fishbone diagram	- Turnaround time (reduced by 49%)	Clinical Laboratory	Case Study
26	“Reducing turnaround time for routine outpatient biochemical tests through Lean Six Sigma: A case study in China” [92]	2024	- DMAIC - VSM - VOC - Pareto analysis	- Turnaround time (reduced by 59%)	Clinical Laboratory	Control Phase
27	“Reducing turnaround time in a pathology laboratory using the lean methodology.” [31]	2023	- Process flowcharts - Fishbone diagram - Kaizen - A3 - Spaghetti diagram	- Turnaround time (reduced from 9.7 to 9 h)	Pathology Laboratory	Pilot
28	“The Impact of Lean in Medical Laboratory Industry: Empirical Studies in Namibia” [54]	2017	- SOP - Root cause analysis - Visual management - 5S - PDCA - Kanban - Value Stream Map	- Turnaround time - Quality improvement - Market share - Cost reduction - Reduced waste (not specified)	Clinical Laboratory	Empirical Study
29	“The Optimization of Total Laboratory Automation by Simulation of a Pull-Strategy” [51]	2015	- Value Stream Map	- Turnaround time (54.5% time saving)	Clinical Laboratory	Case Study
30	“The role of the clinical laboratory in the future of health care: Lean microbiology” [68]	2014	- PDCA - 5S	- Number of corrected reports. - Turnaround time (not specified)	Microbiology Laboratory	Empirical Study
31	“Using Lean Six Sigma to improve timeliness of clinical laboratory test results in a university hospital in Egypt” [93]	2022	- DMAIC - Spaghetti diagram - Why-why	- Turnaround time (not specified)	Hematology Laboratory	Control Phase
32	“Vision of a Microbiology Laboratory of Excellence using Lean” [58]	2018	- Seven wastes - DMADV - SIPOC - Process map - Fishbone diagram - CTQ - Value Stream Map - Pareto diagram	- Delay of results - Waiting time - Physical space needs (not specified)	Microbiology Laboratory	Final Implementation
33	“Workflow optimization in a clinical laboratory using Lean management principles in the pre-analytical phase” [32]	2021	- Lean philosophy	- Turnaround time (reduction of 13%)	Clinical Laboratory	Pilot
