# Supplementary material for: Integrating Lean Six Sigma into Microbiology Laboratories: Insights from a Literature Review
Source: Healthcare (Basel). 2025 Apr 16;13(8):917. doi: 10.3390/healthcare13080917 (PMC12026800; doi:10.3390/healthcare13080917)
Supplement: Supplementary file 1 [file healthcare-13-00917-s001.zip › healthcare-3521564-supplementary.pdf]

CASP
Critical Appraisal
Skills Programme

**CASP Checklist:** 10 questions to help you make sense of a **Systematic Review**

**How to use this appraisal tool:** Three broad issues need to be considered when appraising a systematic review study:

- Are the results of the study valid? (Section A)
- What are the results? (Section B)
- Will the results help locally? (Section C)

The 10 questions on the following pages are designed to help you think about these issues systematically. The first two questions are screening questions and can be answered quickly. If the answer to both is "yes", it is worth proceeding with the remaining questions. There is some degree of overlap between the questions, you are asked to record a "yes", "no" or "can't tell" to most of the questions. A number of italicised prompts are given after each question. These are designed to remind you why the question is important. Record your reasons for your answers in the spaces provided.

**About:** These checklists were designed to be used as educational pedagogic tools, as part of a workshop setting, therefore we do not suggest a scoring system. The core CASP checklists (randomised controlled trial & systematic review) were based on JAMA 'Users' guides to the medical literature 1994 (adapted from Guyatt GH, Sackett DL, and Cook DJ), and piloted with health care practitioners.

For each new checklist, a group of experts were assembled to develop and pilot the checklist and the workshop format with which it would be used. Over the years overall adjustments have been made to the format, but a recent survey of checklist users reiterated that the basic format continues to be useful and appropriate.

**Referencing:** we recommend using the Harvard style citation, i.e.: *Critical Appraisal Skills Programme (2018). CASP (insert name of checklist i.e. Systematic Review) Checklist. [online] Available at: URL. Accessed: Date Accessed.*

©CASP this work is licensed under the Creative Commons Attribution – Non-Commercial-Share A like. To view a copy of this license, visit http://creativecommons.org/licenses/by-nc-sa/3.0/ www.casp-uk.net

CASP
Critical Appraisal Skills Programme

**Paper for appraisal and reference:** Integrating Lean Six Sigma in Microbiology Laboratories: In-sights from a Literature Review

| Section A: Are the results of the review valid? |
|---|

1. Did the review address a clearly focused question?

| | |
|---|---|
| Yes | X |
| Can't Tell | |
| No | |

HINT: An issue can be 'focused' In terms of
- the population studied
- the intervention given
- the outcome considered

| Comments: |
|---|
| The article presents four clearly defined research questions, outlining both the rationale behind their formulation and the current state of the art. It also identifies future research needs based on the issues raised by these questions |

2. Did the authors look for the right type of papers?

| | |
|---|---|
| Yes | X |
| Can't Tell | |
| No | |

HINT: 'The best sort of studies' would
- address the review's question
- have an appropriate study design (usually RCTs for papers evaluating interventions)

| Comments: |
|---|
| The authors have identified available literature on the topic. However, due to the scarcity of articles directly addressing the issue under analysis, LSS projects developed for microbiology laboratories, they have included studies from related fields, such as clinical laboratory research in pathology, biochemistry, and others. This broader search strategy has helped expand the information relevant to the questions raised. |

| Is it worth continuing? |
|---|

3. Do you think all the important, relevant studies were included?

| | |
|---|---|
| Yes | X |
| Can't Tell | |
| No | |

HINT: Look for
- which bibliographic databases were used
- follow up from reference lists
- personal contact with experts
- unpublished as well as published studies
- non-English language studies

| Comments: |
|---|
| The authors conducted a search across major scientific article databases, including Web of Science, ScienceDirect, Scopus, ProQuest, PubMed, and Google Scholar. Additionally, they reviewed the citations of the selected articles to ensure that all relevant available literature was analyzed. Engineering experts in continuous improvement methodologies and lean six sigma were contacted. Clinicians belonging to |

CASP Critical Appraisal Skills Programme

the Microbiology unit of Hospital Universitario Miguel Servet, a tertiary level hospital in Zaragoza (Spain) worked in the study.

https://sectorzaragozados.salud.aragon.es/conocenos/asistencia/mi-hospital/

4. Did the review's authors do enough to assess quality of the included studies?

| | |
|---|---|
| Yes | X |
| Can't Tell | |
| No | |

HINT: The authors need to consider the rigour of the studies they have identified. Lack of rigour may affect the studies' results ("All that glisters is not gold" Merchant of Venice – Act II Scene 7)

**Comments:**
The authors have assessed the suitability of the process analysis tools used in the articles, as well as the consistency of the optimized KPIs, to select the studies that met certain minimum quality criteria. This analysis is also presented in a table attached to the article.

5. If the results of the review have been combined, was it reasonable to do so?

| | |
|---|---|
| Yes | X |
| Can't Tell | |
| No | |

HINT: Consider whether
- results were similar from study to study
- results of all the included studies are clearly displayed
- results of different studies are similar
- reasons for any variations in results are discussed

**Comments:**
The results were grouped for statistical analysis to identify trends over time, similarities between laboratories, and parallels between studies with the same objective. Additionally, differences were analyzed, with particular emphasis on aspects such as the presence of the control phase and its inclusion in a DMAIC strategy.

## Section B: What are the results?

6. What are the overall results of the review?

HINT: Consider
- If you are clear about the review's 'bottom line' results
- what these are (numerically if appropriate)
- how were the results expressed (NNT, odds ratio etc.)

**Comments:**

The study confirmed the growing use of Lean Six Sigma techniques in healthcare and highlighted the most used tools and KPIs. It found that most KPIs are adapted from manufacturing, with patient impact rarely measured, mostly focusing on waiting times or hospital stays, but not clinical aspects like mortality. Only 24% of articles use the control phase, and not always within a DMAIC framework, suggesting areas for future research. Additionally, the inclusion of clinical parameters in Lean Six Sigma optimization and the management of interruptions points to further research needs.

7. How precise are the results?

HINT: Look at the confidence intervals, if given

**Comments:**
Confidence interval analysis is not applicable to the review conducted. This is because the studies included were qualitative, focusing on process optimization, and the review primarily served as a meta-analysis. The statistical analyses in the review were centered around the KPIs and tools identified in the studies, rather than directly analyzing the data extracted from them.

## Section C: Will the results help locally?

8. Can the results be applied to the local population?

| | |
|---|---|
| Yes | X |
| Can't Tell | |
| No | |

HINT: Consider whether
- the patients covered by the review could be sufficiently different to your population to cause concern
- your local setting is likely to differ much from that of the review

**Comments:**
The results have been analyzed from the perspective of applying them to a microbiology laboratory. However, most of the proposed improvements could be applicable to other types of laboratories with minor modifications, or even directly. Furthermore, some of the topics addressed in the review are not covered in other areas of process optimization applied to healthcare, making these suggestions potentially applicable on a broader scale.

9. Were all important outcomes considered?

| | |
|---|---|
| Yes | X |
| Can't Tell | |
| No | |

HINT: Consider whether
- there is other information you would like to have seen

**Comments:**
An evaluation has been conducted from the perspective of bibliographic research on process analysis in general, healthcare in particular, and finally applied to laboratories. Therefore, the potential effects at different levels have been considered. Additionally, both the rationale for formulating the questions and the responses to them are supported by clinical literature

10. Are the benefits worth the harms and costs?

| | |
|---|---|
| Yes | X |
| Can't Tell | |
| No | |

HINT: Consider
- even if this is not addressed by the review, what do **you** think?

Comments:
The benefits are consistently justified from the perspective of improving patient outcomes. The focus is on process optimization, which maximizes resource use while ensuring the reliability of results through clinical parameters, all within the shortest time possible. Additionally, improving laboratory processes has other positive impacts, such as faster responses and the reduction of disease complications caused by delays in identification, among others
